# Environmental exposures and fetal growth: the Haifa pregnancy cohort study

**DOI:** 10.1186/s12889-018-5030-8

**Published:** 2018-01-12

**Authors:** Rachel Golan, Itai Kloog, Ronit Almog, Anat Gesser-Edelsburg, Maya Negev, Maya Jolles, Varda Shalev, Vered H. Eisenberg, Gideon Koren, Wiessam Abu Ahmad, Hagai Levine

**Affiliations:** 1 Ben-Gurion University of the Negev, P.O. Box 653, 84105 Beer-Sheva, Israel; 2 Rambam Hospital, Haifa, Israel; 3 University of Haifa, Haifa, Israel; 4 Institute of Research and Innovation, Maccabitech, Tel-Aviv, Israel; 5 Sackler Faculty of Medicine, Tel Aviv University, Tel-Aviv, Israel; 6 Hadassah-Hebrew University, Jerusalem, Israel; 7 The Hebrew University Center of Excellence in Agriculture and Environmental Health, Jerusalem, Israel

**Keywords:** Environmental exposures, Air pollution, Fetal growth, Indoor air pollution, Pregnancy cohort, Satellite based models

## Abstract

**Background:**

The developing fetus is susceptible to environmental insults. Studying the effects of environmental exposures on fetal growth is essential for understanding the causal pathway between prenatal exposures and pregnancy outcomes. Here we describe the Haifa Pregnancy Cohort Study (HPCS) and discuss challenges and opportunities in applying “big data” paradigm.

**Methods:**

Maccabi Healthcare Services (MHS), is the second largest Israeli health maintenance organization (HMO) providing care services to two million beneficiaries. The HPCS cohort potentially includes ~750,000 newborns born between 1998 and 2017. We will estimate daily exposures to air pollutants, temperature and greenness, using satellite-based data and models. We hypothesize that residents of Haifa have higher exposures to environmental pollutants and that in pregnant women this higher exposure is associated with poorer fetal growth. We will evaluate outcomes such as birth-weight, head-circumference and gestational age at birth. We will adjust for pregnancy complications such as pre-eclampsia and gestational diabetes and parental variables, such as maternal weight, age and smoking habits as potential confounders. In addition, we will conduct a multi-tiered field study, nested within this population, among 150 pregnant women residing in two geographical regions-one in the polluted Haifa area, and one in a relatively unpolluted area in central Israel. Blood and urinary samples will be collected, as well as personal and indoor exposure to air pollution.

**Discussion:**

Evaluating environmental exposures of pregnant women and assessing in utero growth over the course of the pregnancy during different exposure windows, is of great scientific and public health interest. Recent advances in data collection and analysis pose great promise to provide insights into contribution of environment to the health of the developing fetus, but also pose major challenges and pitfalls, such as data management, proper statistical framework and integration of data in the population-based study and selectiveness in the nested field study. Yet the continuing follow-up of the study cohort, integrating data from different services, health-promotion, and eventually, application later in real life of our main promises. Our study aims to meet these challenges and to provide evidence of the environmental exposures associated with fetal growth.

## Background

Fetal development is a global public health concern since in utero growth is suggested as a critical predictor of perinatal and postnatal health and mortality [1, 2]. The developing fetus has been particularly shown to be susceptible to environmental insults [3]. Studying the effects of environmental exposures such as air pollution, temperature and greenness on fetal growth is the necessary first step in defining the causal pathways between prenatal exposures and pregnancy outcomes. These include gestational duration, fetal growth [4], pregnancy loss and congenital anomalies [5, 6], as well as adverse health effects later in life such as childhood obesity [7], cardiovascular disease [8] and respiratory morbidity [9]. Moreover, there are several known determinants of fetal growth that could confound, modify or mediate the association between environmental exposures and fetal growth, such as maternal smoking, socio-demographic status, pregnancy complications and parental characteristics. Presently, evidence of exposure effects during *specific* prenatal periods is still inconclusive [6]. Most studies rely on assessment at birth, yet these do not adequately capture the timing of in utero growth patterns over the course of the pregnancy. Hence, the ability to determine the age at which fetal growth failure begins is suboptimal. Since exposures during early pregnancy may affect fetal growth differently than exposures in later pregnancy [10, 11], identifying exposure windows within pregnancy is critical in the investigation of the pathways leading to these adverse outcomes [12].

Exposure assessment is a major challenge and possible pitfall in environmental epidemiology studies [13]. Many studies rely on a limited number of monitors in their study regions that may provide useful information at the population level, but may also introduce exposure error, and likely biases that affect estimates downward [14]. Broad spatial coverage, enabled by satellites and reliable repeated measurements, allows us to expand exposure data far beyond the range of conventional ground monitoring, particularly for areas and exposure scenarios where surface monitors are not available. This greatly enhances the ability to estimate subject-specific ambient exposures. As previously documented by our group, by using satellite based hybrid PM estimation models [15, 16] we can significantly reduce exposure bias and reliably assess short and long-term human ambient exposures in order to investigate both the acute and chronic effects of ambient particles, respectively. The Haifa Bay is congested with industrial activity. Yet a comprehensive evaluation of exposure of Haifa residents to environmental pollutants is not available.

Maccabi Healthcare Services (MHS) is the second largest Israeli health maintenance organization (HMO) providing primary care services through approximately 3000 health care centers to two million beneficiaries (~25% of the Israeli population) throughout the country. Importantly, MHS has a very low attrition rate at 1% per year. Since the 1990s MHS has been the first fully computerized organization in Israel. Information including patient records, billing systems, pharmaceutical dispensations, and chronic disease registries is housed in the organization’s central electronic warehouse and is available to Maccabi Institute of Research and Innovation. During the physician/nurse patient encounter, all of the information collected is recorded electronically in either coded or free text fields. We designed the Haifa Pregnancy Cohort Study (HPCS) to achieve two goals: (i) Examine whether differences in environmental exposures during different stages of pregnancy are independently associated with fetal growth, controlling for a plethora of personal, obstetrical, medical and sociodemographic characteristics of both parents in a large population of pregnant women and (ii) Evaluate and compare multiple environmental exposures including human biomonitoring and personal exposure to air pollution in a nested panel of 150 pregnant women, members of MHS. We hypothesize that residents of Haifa have higher exposure to environmental pollutants and that in pregnant women this higher exposure is associated with inhibition of fetal growth.

## Methods

### Study population

Maccabi Healthcare Services (MHS), the second largest Israeli health maintenance organization (HMO) provides primary care services to two million beneficiaries (~25% of the Israeli population). Since 1998, information on all members’ interactions with MHS has been collected, including diagnoses, visits to primary and secondary care physicians, visits to outpatient clinics, hospitalizations, laboratory tests, and prescribed and purchased medications. The HPCS cohort potentially includes ~750,000 newborns born between 1998 and 2017 to MHS members.

### Exposure assessment

**Fig. 1:**
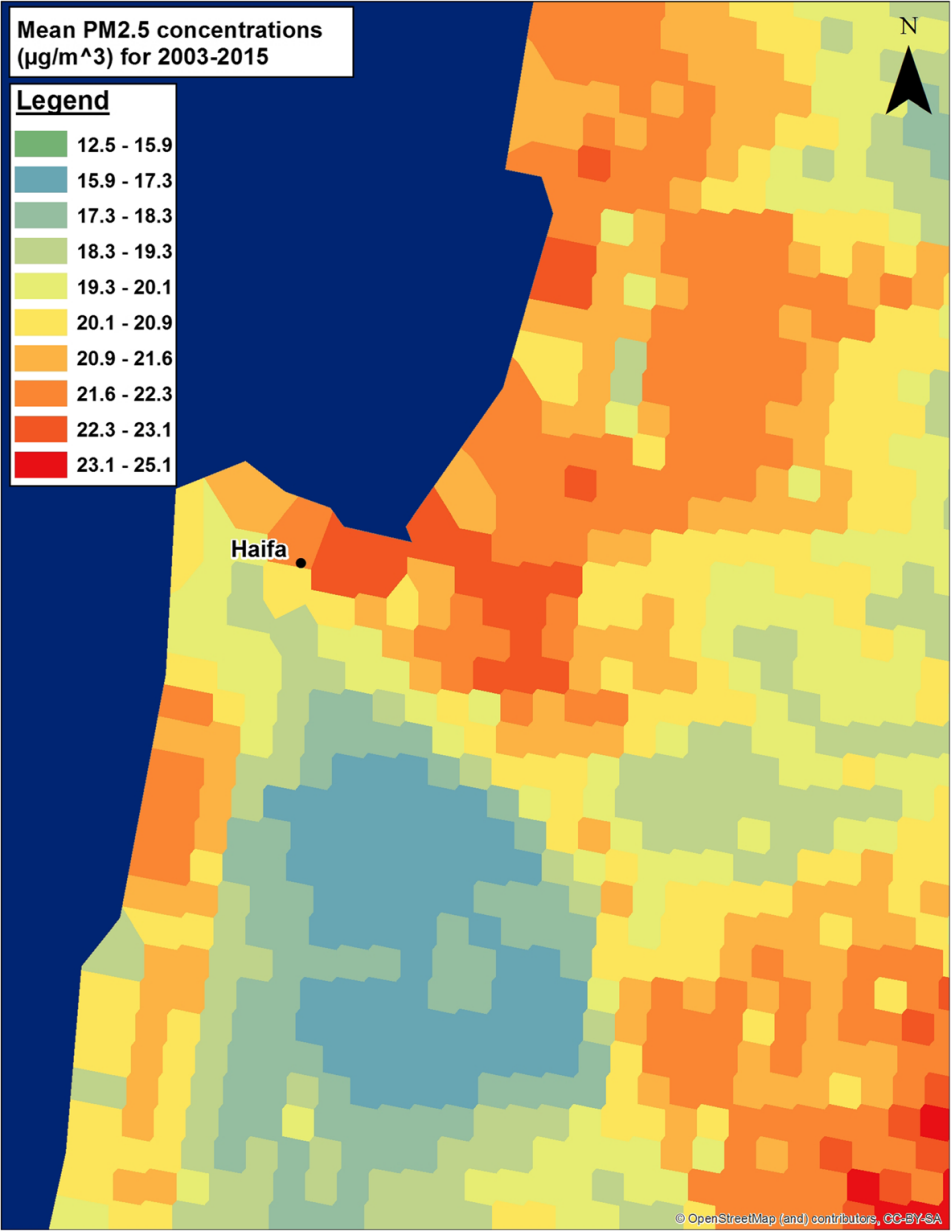
PM2.5 prediction map for Haifa for years 2003–2015 (original data)

We will estimate daily exposures to air pollutants, ambient temperature and greenness (NDVI), using satellite based data and models. PM2.5/10 estimations will be based on novel hybrid satellite based spatiotemporal resolved models developed by us [15–18] for predicting PM10_,_ PM2.5 and temperature (Fig. 1). Our PM models produce high temporal (daily) and spatial (1 square km) resolution PM2.5/10. The exposure model was rigorously validated using standard validation methods such as “ten-fold” out of sample cross validation techniques. We will estimate daily 1 km PM_2.5_ concentration levels for all grid cells in the study including the challenging to model Haifa region. We will take into account the specific geo-topography by using very high resolution elevation and meteorological variables (such as wind) in model calibrations in addition to running several sensitivity analysis taking into account topography barriers. To allow even greater spatial variability at higher resolutions, we will use small-scale spatial predictors defined around each monitoring station, regressing them on the residuals of the third stage model, by use of machine learning techniques. This generates additional daily 200 m “local PM” estimates which are the difference of the estimated local pollution from the average 1 km PM2.5 concentrations at a very fine resolution (200 × 200m). We also developed similar novel satellite based (MODIS) models to estimate air temperature (Ta) at a very fine spatial resolution across Israel [17]. We developed spatiotemporally resolved models which allow us to predict three daily parameters: Ta Max (day time), 24 h mean, and Ta Min (night time) on a fine 1 km grid across the state of Israel. We used and compared both the Aqua and Terra MODIS satellites. We used linear mixed effect models, IDW (inverse distance weighted) interpolations and thin plate splines (using a smooth nonparametric function of longitude and latitude) to first calibrate between Ts and Ta in those locations where we have available data for both and used that calibration to fill in neighboring cells without surface monitors or missing Ts. Out-of-sample ten-fold cross validation (CV) was used to quantify the accuracy of our predictions. Our model performance was excellent for both days with and without available Ts observations for both Aqua and Terra (CV Aqua R2 results for min 0.966, mean 0.986, and max 0.967; CV Terra R2 results for min 0.965, mean 0.987, and max 0.968). The model allows us to generate daily min, mean and max Ta which can be reliably used with high accuracy in Epidemiology studies.

Exposure to green and natural areas around each address will be estimated using a satellite-image based vegetation index. Chlorophyll in plants strongly absorbs visible light (0.4–0.7 μm) for use in photosynthesis, while leaves strongly reflect near-infrared light (0.7–1.1 μm). The Normalized Difference Vegetation Index (NDVI) calculates the ratio of the difference between the near-infrared region and red reflectance to the sum of these two measures, and ranges from −1.0 to 1.0, with larger values indicating higher levels of vegetative density. We will then use GIS analysis to link the NDVI data to each address as a measure of exposure to “green” surroundings as commonly done previously. High-resolution satellite data can be used to accurately assess artificial LAN as it provides precise data at high resolutions (~0.6 km2). LAN data on ambient outdoor night-time illumination for the study area and corresponding years will be obtained from two sources: the radiance-calibrated satellite images of the US Defense Meteorological Satellite Program (DMSP) and the new Visible Infrared Imaging Radiometer Suite Day-Night-Band (VIIRS-DNB) program with higher resolutions than DMSP.

### Outcome

Our MHS database, together with additional data from the Ministry of Health includes demographic data of all members, including newborns, their mothers and fathers, data on birth weight, gestational age at delivery and congenital malformations. Also, we will account for pregnancy complications such as pre-eclampsia, gestational diabetes and hypertension and parental variables, such as maternal weight, BMI, height, age, gravidity, parity, smoking habits and socio-economic parameters as potential confounders. We will evaluate associations between growth outcomes and environmental exposures as mentioned above, for the entire population of pregnant women treated by MHS.

### Nested field study

For the field study, we will recruit 150 pregnant women, members of MHS, with singleton pregnancies as early as possible in pregnancy, at the first visit to gynecologist by week 8 (calculated from the date of the last menstrual period). Inclusion criteria will include nonsmoking women, 18–40 years of age, who are free from chronic diseases such as diabetes and hypertension, who plan to give birth in a hospital and are residents of urban localities in the Haifa bay area and the Rehovot area located in the Central District of Israel. The Rehovot area was chosen based on lower expected exposure to air pollution originating from industrial sources while comparable in socio-demographic characteristics and access to healthcare in a population residing in urban areas of similar magnitude, with similar proportion of MHS. Exclusion criteria will include pregnancy achieved with the aid of fertility treatments, twin or multiple pregnancies and women exposed to second hand smoking at their residency. Recruitment will take place at MHS pregnancy follow-up clinics through a targeted campaign facilitated by the Maccabi clinical research unit and guided by health promotion and health and risk communication experts within our research staff.

### Exposure assessment: Human bio-monitoring

Urine, serum and blood samples will be obtained from the 150 pregnant women three times, in each trimester of pregnancy. Samples are planned to be examined for: (1) VOCs including blood VOCs, serum aldehydes, urinary VOCs metabolites, aromatic diamines (and creatinine); (2) Elements using ICP-MS sensitive methods 3) Hydroxy polycyclic aromatic hydrocarbons using HPLC-MS/MS sensitive methods [19] and 4) cotinine using sensitive GC-MS method. In addition, the stored bio-samples will serve as infrastructure for measuring further exposures in the future, including non-targeted approaches.

### Indoor air pollution and personal exposure

Indoor air will be sampled at residencies of the 150 pregnant women living in Haifa and Rehovot at the same time biological samples will be collected. A sampler will be placed in the main activity room (other than kitchen). Sampling will include measurements of PM2.5 and nitrogen dioxide (NO2), for two consecutive days. Women will complete a questionnaire providing information about the type, age, and size of the home, as well as information about indoor sources that may impact air quality measurements, such as cigarettes, wood stoves, and candles. Participants will keep a log of open window status and heating, ventilation and air conditioning (HVAC) use during the sampling period. In addition, women will carry a personal air monitor that will sample personal exposure to PM2.5 and NO2. Participants will complete a daily time-activity questionnaire regarding time spent indoors, outdoors or in transit and additional data regarding life-style, occupational and dietary exposures. Anthropometric measurements will be taken. A research nurse and an assistant will visit the homes, while minimizing participant burden. Whenever possible, we will use similar questionnaires and bio-samples collection protocols as part of the Israeli Birth Cohorts Consortium. Harmonization will allow comparison and data pooling with other studies conducted in Israel [20], which is especially important in a small country with limited resources.

Also, we will assign ambient exposure on our well-established exposure assessment models and the geocoded location of the participants’ residence. This will yield spatiotemporally resolved robust ambient estimates.

### Ethical consideration

The Helsinki committee of Assuta Medical Center approved the study protocol. For the retrospective study, the committee granted exemption from informed consent since it includes de-identified electronic medical records. For the field study, informed consent will be obtained from all women upon recruitment.

### Statistical analysis

Data analysis will be conducted using IBM SPSS STATISTICS software, version 24.0 and STATA software, version 14.0. The distribution of the continuous variables will be checked by the skewness using the Cox test (coefficient of skewness divided by standard error of skewness) as well as examination of the mean–median difference and the Q-Q plot. Skewed data will be log-transformed. Chemical measures in the biomonitoring, will be described by medians, inter-quartiles range, geometric means and maximum values for analyte concentrations. Correlations between exposures will be assessed using Spearman correlations tests, overall and by sub-groups. We will compare exposures by geographical regions, first by comparing means and proportions and later by using univariate and multivariate quantile or logistic regression models. Associations between environmental exposures and fetal growth will be analyzed using advanced statistical methods, used by our group in other studies [20, 21].

We will use linear mixed-effect regression models to estimate associations between environmental exposures during different time windows and growth continuous outcomes (birth weight and head circumference), and logistic mixed-effect regression models to estimate associations with preterm birth (< 37 weeks), low birth weight (LBW) (< 2500 g) or microcephaly. Specifically, we will fit the following models:

1 $$ {BW}_{ij}=\left(\alpha +{u}_j\right)+{\beta}_1{PM}_i+{\beta}_{2i}{X}_{2i}+{\beta}_{3i}{X}_{31}\dots +{e}_{ij}\ \left({u}_j\right)\sim N\left[0,{\sigma_u}^2\right] $$

2 $$ Logit\left({PT}_{ij}/{LBW}_{ij}=1|X\right)=\left(\alpha +{u}_j\right)+{\beta}_1{Ta}_i+{\beta}_2{PM}_i+{\beta}_3{X}_{3i}+{\beta}_{4i}{X}_{4i}\dots +{e}_{ij}\ \left({u}_j\right)\sim N\left[0,{\sigma_u}^2\right] $$

where BW_ij_/*PT*_*ij*_*/LBW*_*ij*_ is the response (birth weight*/*logistic outcomes- preterm, LBW etc) for the *i*th subject in small statistical area *j*, *α* and *u*_*j*_ are the fixed and random (area specific) intercepts, respectively*,* PM_i_, X_1i_, etc. denote the set of covariates of interest used in the model, e_ij_ is the error term and finally, *σ*_*u*_^*2 i*^is the variance of the tract random effects, and *e*_*jj*_*~ N[0,σ*_*e*_^*2*^*].*

Gestational duration and prematurity will be also analyzed using a Cox proportional hazards model, with gestational age as the basic time scale and adjusted for the same factors as the anthropometric measurements. Linearity of the relationship between the outcomes under study and PM_2.5_ and additional exposures will be explored using smooth functions, such as splines. We will further examine interactions between PM_2.5_ and other potential predictors such as high ambient temperatures.

## Discussion

Evaluating environmental exposures of pregnant women and assessing in utero growth over the course of the pregnancy during different exposure windows, is of great scientific and public health interest. Recent advances in data collection and analysis pose great promise to provide insights into to contribution of environment to the health of the developing fetus, but also pose major challenges and pitfalls. Our study design, detailed here, aims to meet the challenges and to provide evidence regarding the actual environmental exposures of residents in Haifa, in comparison to other areas in Israel, and its association with fetal growth. Methodology developed may aid further research and findings may inform policy, as well as add to the growing body of literature regarding the complex biological effects of environmental exposures in early life.

Our study has several limitations that warrant consideration. The data used in this study were primarily recorded for administrative purposes. Although methods for data collection have improved along the years, the methods of collecting information on variables such as BMI and smoking may vary among medical personnel within the same HMO, which might lead to missing information. Strengths of our study include the use of state-of-the-art methods to assess multiple environmental exposures, the comprehensive computerized database across 20 years on a large population of potentially 750,000 newborns and the nesting of the field study within the same population-based study.

The field study will provide evidence regarding the actual exposure to multiple chemicals of residents in Haifa, in comparison to other areas and the possible association to fetal growth. Since the prenatal cohort study is nested within the entire population of the MHS, the framework established will serve as a health data infrastructure that could be utilized to avoid the pitfalls of both small-scale study with direct measurement of exposure, and of a large-scale study with exposure assessment based on place of residence. We will be able to assess the representativeness of the study participants in the field study using data from the MHS databased. We will use exposure data from the field study to inform the modeling of the larger study. The unique setting provides us with the opportunity to examine these questions in far more detail and accuracy than before.

There are still some major challenges to tackle in such big data study. The wealth of data on each individual may risk the de-identification process and requires expertise in data management in order to ensure the privacy of the participants. The high volume of data allows endless possibilities for statistical analysis and warrant a hypothesis driven predetermined statistical framework in order to avoid incidental findings.

The analysis of big data through models such as machine learning, provides a unique opportunity to identify trends and findings which are not possible to figure out through classical statistical models. Last year MHS inaugurated a new research group within our Research Institute fully dedicated to Big Data analysis (The Morris Kahn –Maccabi Data Science Institute). Members of The Haifa Pregnancy Cohort Study are scientists in this new research group, and the platform, tools and staff will be available to aid in the analysis of the environmental data.

## Abbreviations

HMO: Health Maintenance Organization
HPCS: Haifa Pregnancy Cohort Study
MHS: Maccabi Healthcare Services
